# Dietary behaviour is key to human and planetary health

**DOI:** 10.1017/S1368980024000594

**Published:** 2024-03-20

**Authors:** Charlotte Elizabeth Louise Evans

**Affiliations:** Purple Nutrition, 17 Hazel Drive, Chesterfield, S40 3EN, UK

**PHN editorial Feb 2024**

## Improving dietary behaviour is key to planetary health as well as human health

It has now been a year since I took on the role of editor in chief at the *Public Health Nutrition* (*PHN*) journal and what an eventful year it has been. The first few months were spent getting to grips with the nuts and bolts of the journal and the systems in place for authors and reviewers. Despite working for many years as an associate editor for *PHN*, there was still much to learn, and I am grateful to Jennifer Lindsay-Smith at Cambridge University Press for her support and patience. This greater understanding has enabled me to start tackling some of the thorny issues facing many journals, including supporting the editorial board in finding suitable reviewers and reducing the length of time to first decision for authors. We know that timeliness in publishing is important to all researchers but should never be at the expense of a rigorous and effective peer review process. We are pleased that we have reduced the time to first decision to just over 2 months and continue to strive to reduce it further. We have also increased the number of editors on the editorial board and aim to increase it further over the next 12 months helping to reduce the workload of individual board members. We are immensely grateful to all the editorial board members for their work on processing manuscripts and contributing to the direction of the journal.

The second half of my first year has been more reflective. Perhaps never before have we faced such unpredictable global challenges; poverty, war and climate crisis are increasingly impacting many people’s daily lives. Food and food systems are at the heart of many of these global issues, and publishing high-quality public health nutrition research has never felt more important. We are in need of a range of innovative solutions to improve nutrition behaviour, and sharing best research and practice is a key focus of this journal. Solutions to improve diet and health and equity must also take into account environmental factors to protect the planet, not just in terms of carbon footprint but also water footprint and impact on biodiversity. There is a long way to go to ensure that we do not experience ecological overshoot (consuming above the planet’s capacity). A recent article highlighted that successfully tackling the climate crisis over the next decades will be impossible without changing behaviour and changing the drivers of consumers’ behaviour^(1)^. Successful ways of changing relevant food-related behaviours such as reducing meat consumption and reducing food waste (particularly animal-based food waste) are key to tackling the climate crisis. The authors conclude that positive behaviour changes will only happen with an overhaul in the way food (and other products) are retailed, priced and marketed^(1)^, topics which are firmly within the remit of *PHN*.

Priorities for this journal therefore reflect these global challenges and include evaluation of solutions to improve human and planetary health and reduce inequity. Policy-relevant research (including systematic reviews) is key to influencing policy decisions at regional, national and international levels, and therefore, we encourage authors to submit policy-related research to *PHN*. We also encourage commentaries on important topics in addition to original research. These are manuscripts that discuss key global issues and have included papers on a wide range of topics over the last 12 months such as food environments and food systems, childhood obesity and adherence to interventions. To help readers identify relevant papers, we are expanding our online collections that collate relevant published papers from the journal over the previous 5 years. We have recently added collections on sugary drinks and health, plant-based diets and nutrition policy and are soon to add two more on food labelling and food marketing. Many of the papers included are open access.

In summary, it has been an enjoyable year guiding the *PHN* journal on the next stage of its journey. As I look at the list of manuscripts accepted for publication in January and February, I am proud of the sheer diversity of research from many different regions of the world, different stages in the life course and on a range of aspects of public health nutrition. We hope you have enjoyed reading and contributing to the journal and welcome suggestions for future collections. Wishing you a healthy and peaceful 2024.

Dr Charlotte Evans
